# Supplementary material for: Nitration of 2,6,8,12-Tetraacetyl-2,4,6,8,10,12-Hexaazaisowurtzitane Derivatives
Source: Materials (Basel). 2022 Nov 8;15(22):7880. doi: 10.3390/ma15227880 (PMC9692839; doi:10.3390/ma15227880)
Supplement: Supplementary file 1 [file materials-15-07880-s001.zip › materials-2009340-supplementary.pdf]

Supplementary Materials

Nitration of 2,6,8,12-Tetraacetyl-2,4,6,8,10,12-Hexaazaisowurtzitane Derivatives

Maya V. Chikina *, Daria A. Kulagina and Sergey V. Sysolyatin

Laboratory for Medicinal Chemistry, Institute for Problems of Chemical and Energetic Technologies, Siberian Branch of the Russian Academy of Sciences (IPCET SB RAS), 659322 Biysk, Russia

* Correspondence: chikina_maya@mail.ru; Tel.: +7-3854-30-15-45

Figure of Contents

^{1}H,^{13}C NMR spectra for compounds **7** and **8**……………………………...S1−S4

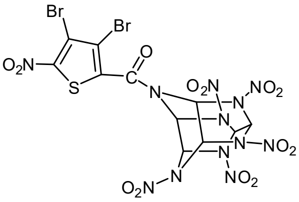

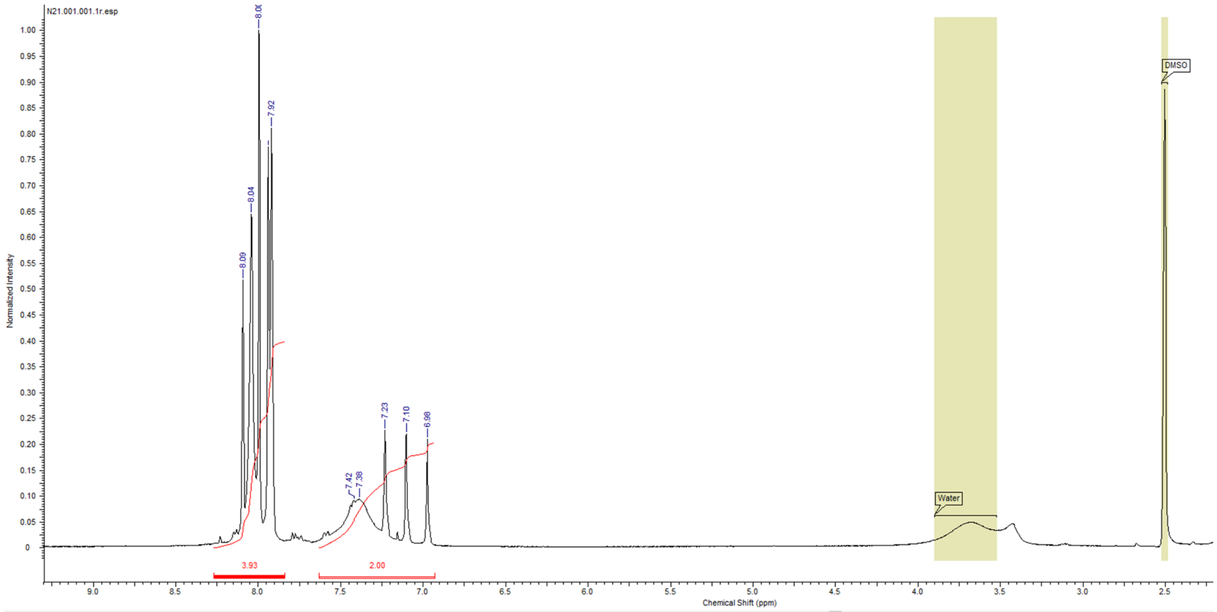

Figure S1. ^{1}H NMR spectrum of 4-(2-Nitro-3,4-dibromothiophenecarbonyl)-2,6,8,10,12-pentanitro-hexaazaisowurtzitane **7** in DMSO-d6

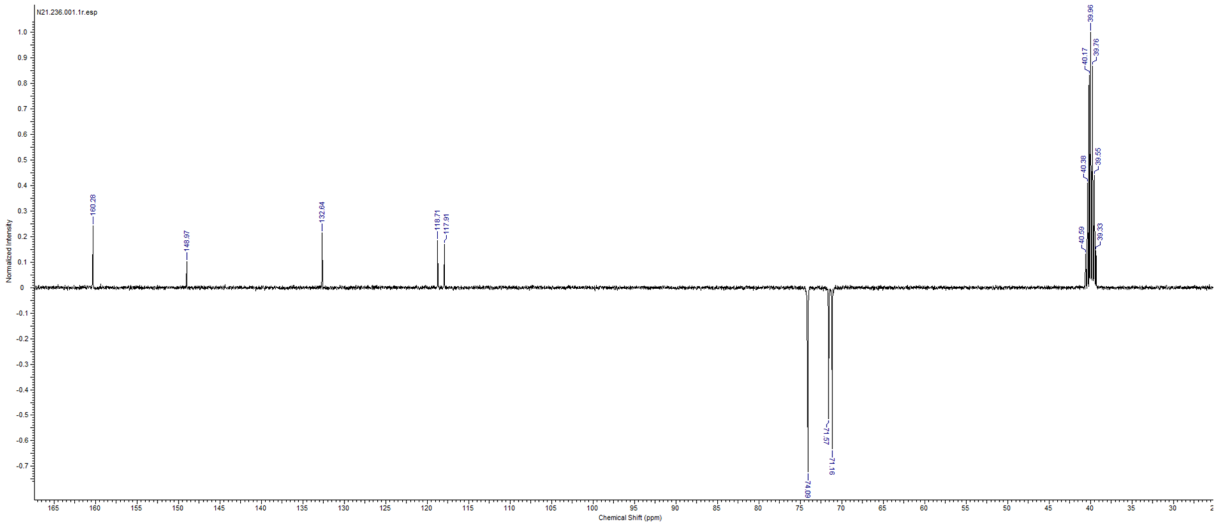

Figure S2. ^{13}C NMR spectrum of 4-(2-Nitro-3,4-dibromothiophenecarbonyl)-2,6,8,10,12-pentanitro-hexaazaisowurtzitane **7** in DMSO-d6

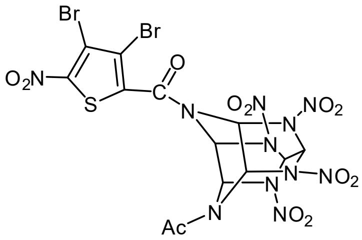

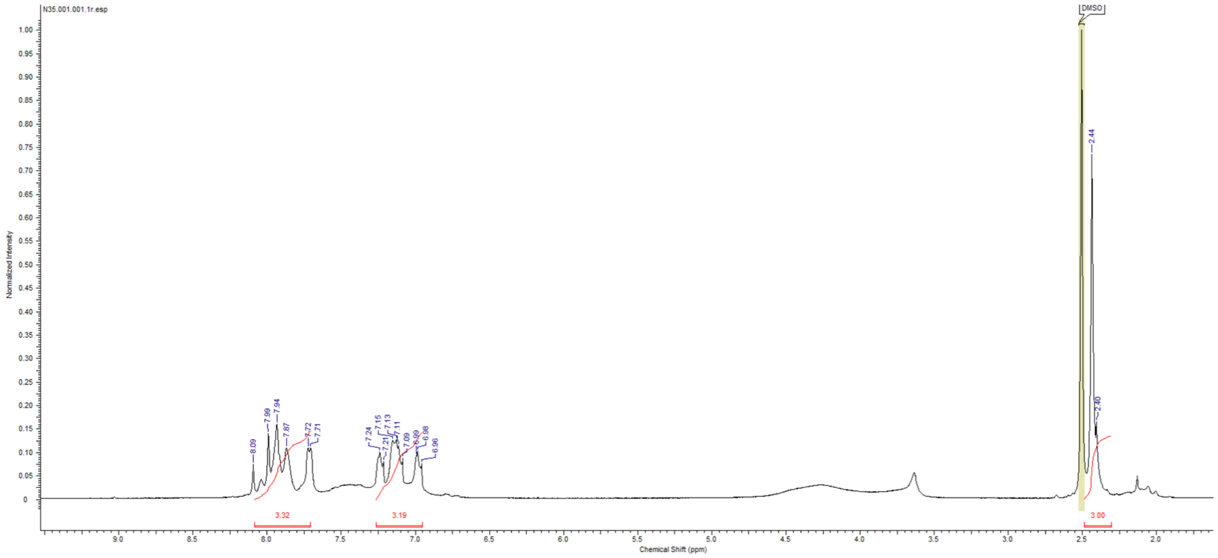

Figure S3. ^{1}H NMR spectrum of 4-(2-Nitro-3,4-dibromothiophenecarbonyl)-10-acetyl-2,6,8,12-tetranitro-2,4,6,8,10,12-hexaazaisowurtzitane **8** in DMSO-d6

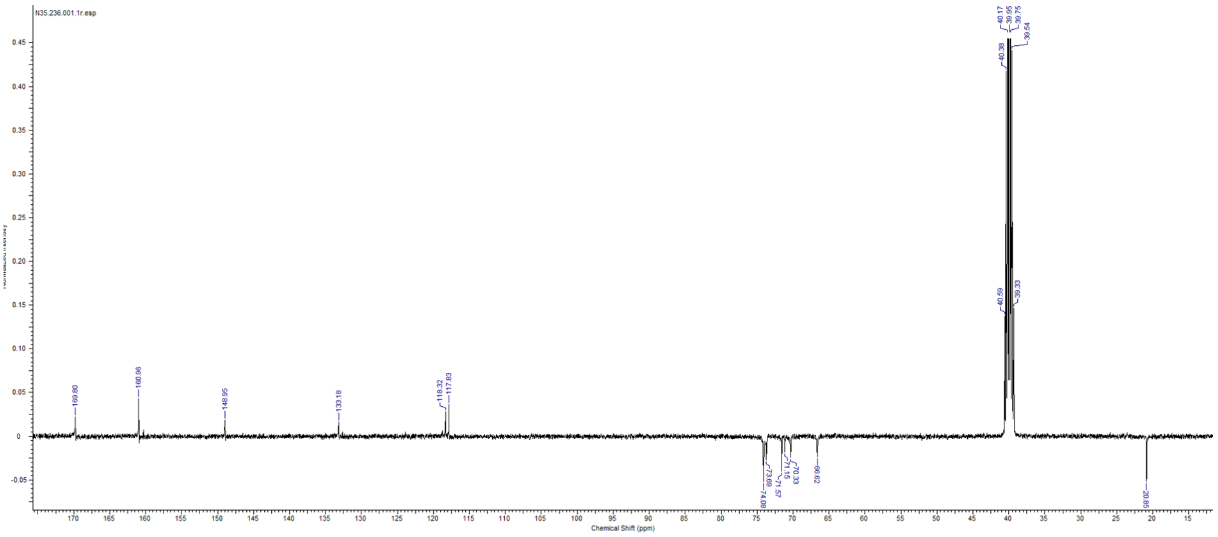

Figure S4. ^{13}C NMR spectrum of 4-(2-Nitro-3,4-dibromothiophenecarbonyl)-10-acetyl-2,6,8,12-tetranitro-2,4,6,8,10,12-hexaazaisowurtzitane **8** in DMSO-d6
